# Chorioamnionitis in the Second Trimester Secondary to Multidrug‐Resistant *Haemophilus influenzae* Infection

**DOI:** 10.1155/crdi/6256725

**Published:** 2026-04-06

**Authors:** Karen S. Carlson, Morgan Baker Korthals

**Affiliations:** ^1^ Department of Obstetrics and Gynecology, College of Medicine, University of Nebraska, Omaha, Nebraska, USA, unl.edu

**Keywords:** chorioamnionitis, intrauterine infection, multidrug-resistant *Haemophilus influenzae*, preterm prelabor rupture of membranes (PPROM), second-trimester pregnancy loss

## Abstract

**Purpose:**

Nontypeable *Haemophilus influenzae* (NTHi) is an emerging perinatal pathogen linked to chorioamnionitis, preterm birth, and early pregnancy loss. This case report describes the presentation, microbiologic findings, and management of second‐trimester NTHi‐associated chorioamnionitis, emphasizing diagnostic challenges and antimicrobial implications.

**Methods:**

A 22‐year‐old woman at 22 weeks 3 days’ gestation presented with preterm prelabor rupture of membranes (PPROM) and vaginal bleeding. Evaluation included cervical examination and bedside ultrasound. Placental tissue was collected for culture and antimicrobial susceptibility testing. The hospital course, clinical interventions, and postpartum outcomes were recorded. A literature review of reported cases was conducted to contextualize the findings and inform management strategies.

**Results:**

On admission, cervical dilation was 3 cm, and fetal heart tones were present. Ultrasound demonstrated anhydramnios. Maternal inflammatory markers were elevated, consistent with chorioamnionitis. Despite the use of antibiotics, spontaneous vaginal delivery occurred at 22 weeks 3 days, complicated by cord avulsion and manual placental extraction. Placental cultures grew NTHi resistant to ampicillin and amoxicillin–clavulanate but susceptible to ceftriaxone, azithromycin, and ciprofloxacin. Postpartum complications included retained products of conception requiring dilation and curettage. Reported cases similarly support ascending genital tract infection as the most likely source and demonstrate increasing antimicrobial resistance in pregnancy‐associated isolates.

**Conclusion:**

NTHi represents an uncommon yet clinically important cause of second‐trimester chorioamnionitis and early pregnancy loss. Clinicians should consider NTHi in cases of intrauterine infection when standard pathogens are not identified or when empiric antibiotic therapy fails. Early recognition, comprehensive microbiologic evaluation, and antibiotic selection guided by susceptibility patterns are essential to optimize maternal and fetal outcomes. Rising antimicrobial resistance among NTHi strains underscores the need for continued surveillance and evidence‐based management strategies in pregnancy.

## 1. Introduction

*Haemophilus influenzae* is a Gram‐negative coccobacillus that colonizes the upper respiratory tract and evades host defenses via IgA protease [1]. It is classified into six encapsulated serotypes and unencapsulated strains. The unencapsulated strains are called nontypeable [2]. Following widespread *Haemophilus influenzae* type b (Hib) vaccination in 1985, nontypeable *Haemophilus influenzae* (NTHi) has become the predominant cause of *H. influenzae* infection, particularly in perinatal infants, young children, and older adults [3, 4].

In adults, NTHi typically causes respiratory infections, while in children, it often presents as otitis media, sinusitis, or pneumonia [5]. Since these nontypeable strains do not have a capsule, they do not usually cause invasive disease. Meningitis, epiglottitis, and sepsis, the more severe and invasive infections, are mainly caused by Hib [2]. Pregnant women are particularly susceptible, with infection rates over five times higher than in nonpregnant women [6]. In addition, NTHi has been associated with early pregnancy loss and preterm birth before 24 weeks [7]. Preterm infants are especially vulnerable, with a 23‐fold increased risk compared to term infants [6].

Clinical chorioamnionitis occurs in approximately 5%–12% of term pregnancies and is usually caused by a polymicrobial infection. Among women with suspected infection, the organisms most frequently identified in amniotic fluid cultures include ureaplasma urealyticum and mycoplasma hominis. In preterm prelabor rupture of membranes (PPROM), chorioamnionitis has been diagnosed in 30% of cases [8]. Intrauterine infection may occur via ascending genital tract spread or maternal bacteremia [9]. Evidence increasingly links NTHi to chorioamnionitis [10, 11], and rising ampicillin resistance complicates empiric treatment [12]. Understanding transmission and resistance patterns is critical to improving maternal and neonatal outcomes.

Chorioamnionitis is most common in the third trimester or during labor, and second‐trimester cases are rare but frequently associated with adverse outcomes, including preterm labor and fetal demise [13]. While typical pathogens include Group B Streptococcus, Escherichia coli, and anaerobes, *H. influenzae* infections are uncommon and may be multidrug‐resistant, which further complicates management. We present a case of second‐trimester chorioamnionitis caused by multidrug‐resistant NTHi with a severe clinical course.

## 2. Case Presentation

### 2.1. Clinical Presentation

A 22‐year‐old G2P0010 woman at 22 weeks 3 days of gestation, based on a 10‐week 3‐day dating ultrasound, presented with PPROM and vaginal bleeding. Her past obstetrical history was significant for an early miscarriage that occurred 4 years prior, for which there was no clinical record available. Her past medical history was significant for anxiety, depression, and post‐traumatic stress disorder. She also had syphilis in this pregnancy, methamphetamine and tobacco use, and unstable housing. Earlier in this pregnancy, the patient was partially treated for syphilis with intramuscular penicillin G. An infectious disease (ID) consultation was requested for a positive fluorescent treponemal antibody‐absorption (FTA‐ABS) test and negative rapid plasma reagin (RPR). This was thought to be consistent with early syphilis infection. Noninvasive prenatal testing (NIPT) was negative, indicating a low risk for trisomy 21, 18, 13, and common sex chromosome abnormalities.

At 15 weeks 4 days, the patient had presented to the emergency department (ED) with light vaginal spotting. At that time, the patient was afebrile with a white blood cell (WBC) count of 11.3 × 10^3^/μL. No etiology for the bleeding was found. At 20 weeks 4 days, a routine anatomy ultrasound showed a thickened placenta (4.8 cm) and intrauterine fetal growth restriction (IUGR) with an estimated fetal weight in the 8^th^ percentile. A maternal‐fetal medicine (MFM) consultation was obtained. The patient was offered an amniocentesis for definitive chromosomal testing, but she declined. A follow‐up ultrasound was planned for 4 weeks.

At 22 weeks 3 days, she presented with PPROM, vaginal bleeding, and contractions. Admission labs showed WBC 23.1 × 10^3^/μL (normal 4.0–11.0 × 10^3^/μL), absolute neutrophil count (ANC) 18.9 × 10^3^/μL (normal 1.3–7.5 × 10^3^/μL), and maximum temperature 37.4°C on that day. The estimated fetal weight was < 450 g. Latency antibiotics were initiated with intravenous ampicillin and erythromycin, per standard PPROM protocol. Despite this, labor progressed, resulting in a spontaneous vaginal delivery at 22 weeks and 3 days.

The infant’s weight was 410 g, and the Apgar scores were 4, 3, and 1 at 1, 5, and 10 min, respectively. Birth length was 10.5 inches, and head circumference was 7.28 inches. The neonatal intensive care unit (NICU) did not resuscitate the baby due to extreme prematurity. The neonate subsequently died. At delivery, placental cultures were obtained from the subchorionic fibrin layer beneath the fetal membranes using a sterile swab technique. The swab was applied to the subchorionic layer just beneath the chorionic plate to evaluate for chorioamnionitis. Postpartum, maternal labs improved: on postpartum Day 1, WBC was 21.5 × 10^3^/μL and ANC 16.7 × 10^3^/μL; on postpartum Day 2, WBC was 14 × 10^3^/μL and ANC 9.3 × 10^3^/μL.

### 2.2. Microbiology and Antibiotic Resistance

• *Haemophilus influenzae*: moderate growth; resistant to ampicillin (MIC ≥ 256), amoxicillin–clavulanate (MIC 16), and trimethoprim–sulfamethoxazole (MIC 4); susceptible to azithromycin (MIC 4), ceftriaxone (MIC 0.25), and ciprofloxacin (MIC 0.016)
• Rare coagulase‐negative *Staphylococcus* species
• Rare *Streptococcus mitis/oralis* (viridans group)
• Few anaerobic Gram‐negative rods (susceptibility not routinely tested)

### 2.3. Hospital Course and Management

Postdelivery, the patient was clinically stable, asymptomatic, and ambulatory. She received intramuscular benzathine penicillin for previously diagnosed but incomplete syphilis treatment. On postpartum Day 1, she experienced persistent vaginal bleeding and passage of clots. Transvaginal ultrasound demonstrated retained products of conception with a 2.1 cm thickened endometrium with increased endometrial vascularity. She underwent dilation and curettage (D&C) under anesthesia after counseling regarding bleeding and infection risks.

Postoperatively, she recovered well and was discharged home without additional postpartum antibiotics, consistent with the American College of Obstetrics and Gynecology (ACOG) recommendations that 24 h of IV antibiotic therapy is sufficient after delivery for chorioamnionitis if the patient is clinically stable. Placental histopathology later demonstrated acute chorioamnionitis (Grade 2/Stage 2) with associated subchorionitis and acute vasculitis of the chorionic plate vessels. The umbilical cord showed acute funisitis with phlebitis and arteritis, representing a fetal inflammatory response.

## 3. Discussion

PPROM occurs in approximately 2%–3% of pregnancies and contributes to nearly one‐third of preterm deliveries. Although empiric antibiotics are commonly used in PPROM, evidence suggests they do not consistently reduce severe neonatal morbidity or perinatal mortality or improve long‐term outcomes. Additionally, the optimal regimen and duration of antibiotic therapy remain unclear. Up to one‐third of patients with PPROM before 34 weeks likely have intraamniotic infection, yet routine amniocentesis for Gram stain and culture is not recommended by international guidelines [14]. Current practice generally follows empiric antibiotic protocols, such as those supported by the ORACLE trial [15]. Emerging evidence indicates that management could be refined by combining empiric coverage with consideration of pathogen virulence and tailoring antibiotic duration based on microbiologic or amniotic fluid analysis when available. This approach aligns with antimicrobial stewardship principles by reducing unnecessary exposure, minimizing disruption of the maternal and neonatal microbiome, and limiting the development of antimicrobial resistance. Targeted therapy may also reduce hospital stay, lower the risk of hospital‐acquired complications, and improve patient outcomes in select low‐risk populations [14].

NTHi is increasingly recognized as an important perinatal pathogen, particularly in early pregnancy loss and preterm birth. Historically, invasive *H. influenzae* infections in pregnancy were rare due to widespread vaccination against serotype b (Hib), which dramatically reduced morbidity and mortality [7, 11]. However, NTHi, which lacks a polysaccharide capsule, has emerged as the predominant cause of invasive disease among vulnerable populations, including pregnant women, neonates, and those with comorbidities [7]. In 2017, the first reported septic abortion from beta‐lactamase‐negative ampicillin‐resistant NTHi was reported in Japan. Before this, NTHi was not considered a causative agent in obstetrical infections [16].

Haemophilus influenzae develops antibiotic resistance primarily through β‐lactamase production and mutations in the ftsI (filamentous temperature‐sensitive) gene that alter penicillin‐binding protein 3. These resistance mechanisms typically arise with repeated or prolonged β‐lactam exposure, particularly in respiratory tract infections, where sustained selective pressure promotes the emergence of resistant strains [4, 17]. In contrast, brief, targeted antibiotic exposure, such as the limited treatment for syphilis with penicillin G in our patient, is unlikely to generate or maintain these resistance patterns.

In this context, our patient presented with preterm PPROM at 22 weeks’ gestation, vaginal bleeding, and clinical signs consistent with intrauterine infection. According to the July 2024 ACOG Clinical Practice Update, suspected intraamniotic infection is identified by maternal fever ≥ 39°C or 38.0°C–38.9°C plus another risk factor and may be diagnosed even in afebrile patients if other clinical features are consistent with infection. Placental cultures from our patient confirmed NTHi, which was resistant to ampicillin and amoxicillin–clavulanate but susceptible to ceftriaxone, azithromycin, and ciprofloxacin. This aligns with reports of rising antimicrobial resistance among NTHi strains due to both β‐lactamase production and horizontal transfer of resistance genes [18, 19]. Recognition of these resistance patterns is critical because delayed or ineffective empiric therapy may exacerbate maternal and fetal morbidity.

In our case, neonatal death was most likely attributable to complications of extreme prematurity in the setting of intraamniotic infection. Placental histopathology demonstrated acute chorioamnionitis (Grade 2/Stage 2), subchorionitis, and chorionic plate vasculitis, with associated funisitis involving both the umbilical vein and arteries, indicating a fetal inflammatory response. These findings are characteristic of intrauterine infection. Maternal inflammatory markers, including elevated WBC and ANC, reflected a significant intrauterine inflammatory response. Although C‐reactive protein (CRP) and other markers such as procalcitonin (PCT) or interleukin‐6 (IL‐6) were not obtained, the trend in WBC and ANC, along with clinical findings, supported the diagnosis of chorioamnionitis.

The isolation of NTHi from placental cultures supports an ascending genital tract infection as the underlying etiology. The resulting intraamniotic infection likely precipitated preterm labor and delivery at a previable gestational age, with neonatal death occurring shortly after birth due to complications of extreme prematurity. In chorioamnionitis, intense maternal‐fetal inflammation may trigger spontaneous preterm birth, prioritizing maternal survival at the expense of fetal viability [20].

Although most prophylaxis and management studies focus on term prelabor rupture of membranes, evidence suggests that combination regimens, such as ampicillin plus gentamicin, reduce rates of clinical chorioamnionitis, maternal postpartum complications, and adverse neonatal outcomes compared with ampicillin alone [8]. While data are limited for second‐trimester PPROM, these findings underscore the importance of reconsidering empiric antimicrobial strategies in high‐risk pregnancies, particularly when multidrug‐resistant pathogens such as NTHi are suspected. Tailoring therapy based on local resistance patterns and individual patient risk factors may therefore improve outcomes. Preterm birth complicated by histologic chorioamnionitis is associated with higher rates of extreme preterm birth and adverse neonatal outcomes in subsequent pregnancies, highlighting the long‐term implications for maternal and infant health [21].

The mechanism of intrauterine NTHi infection is thought to be primarily ascending colonization from the genital tract, supported by reports of maternal vaginal and cervical colonization preceding chorioamnionitis [11]. Genomic studies have shown that microbial populations in the vagina, intrauterine environment, and neonatal circulation are closely related, supporting the hypothesis that ascending infection allows microorganisms to cross maternal–fetal boundaries [22]. Nontypeable isolates from neonatal invasive disease show a predilection for the urogenital tract, and a case series found NTHi to be present on high vaginal swabs in women with early pregnancy loss [14]. Alternatively, hematogenous spread remains an additional plausible route of infection, although less commonly documented. Evidence for this route includes a large surveillance study, which showed that nearly half of pregnant women with invasive *H. influenzae* infection had NTHi bacteremia [7]. Hematogenous spread from the oral cavity represents this biologically plausible but less common route, which has been clearly demonstrated for oral pathogens like Fusobacterium nucleatum [23].

In the setting of the placental histopathologic features and the patient’s clinical presentation with PPROM and preterm labor, the isolation of NTHi from placental cultures is unlikely to represent contamination and instead supports a pathogenic role for NTHi.

Our case highlights the diagnostic challenges of NTHi infection in pregnancy. The organism is fastidious, and routine genital‐tract cultures rarely detect it; thus, targeted microbiologic evaluation of placental and fetal tissues is essential [7]. Early identification can facilitate appropriate empiric antibiotic therapy, which is critical to optimizing maternal and neonatal outcomes.

Although earlier suspicion of NTHi could allow for targeted therapy, our patient presented after PPROM with an established infection, making it unlikely that earlier recognition would have changed the outcome. Initial management was guided by empiric antibiotics for suspected chorioamnionitis. While amniocentesis with Gram stain and culture could potentially identify the pathogen sooner, in this case, it likely would not have altered the clinical course. The patient, unfortunately, went on to quickly deliver a nonviable fetus. Nevertheless, the increasing recognition of NTHi underscores the need for clinical awareness and adjustment of therapy based on culture and susceptibility results, particularly given rising antimicrobial resistance.

In summary, NTHi infection should be considered in pregnant women presenting with vaginal bleeding, abdominal pain, and clinical signs of infection, especially in the context of PPROM or early pregnancy loss. Awareness of emerging antimicrobial resistance, social determinants of health, and potential for severe perinatal complications is essential for clinicians managing these high‐risk pregnancies. Future studies should evaluate population‐level prevalence, optimal screening strategies, empiric therapy, and preventive measures to reduce adverse outcomes associated with NTHi.

### 3.1. Key Clinical Challenges

This case illustrates several key clinical challenges:

• Early‐onset chorioamnionitis in the second trimester is uncommon and often portends poor fetal outcomes.
• Infection with Haemophilus influenzae, a rare cause of chorioamnionitis, demonstrated multidrug resistance, including resistance to Ampicillin and Augmentin, complicating empiric antibiotic choices.
• The patient’s social determinants, including substance use and unstable housing, likely contributed to infection risk and complicated postdelivery care.

## 4. Conclusion

This case underscores the importance of considering uncommon and antimicrobial‐resistant pathogens, such as NTHi, in chorioamnionitis, particularly in high‐risk pregnancies complicated by PPROM. Prompt recognition, targeted microbiologic evaluation, and tailored antimicrobial therapy are essential to optimize outcomes. Multidisciplinary management that addresses both medical and social complexities, including substance use, housing instability, and comorbidity, are critical in achieving safe and effective care. A clearer understanding of transmission pathways may also help to guide prevention strategies and improve early recognition of intraamniotic infection. Given the emerging prevalence and evolving resistance patterns of NTHi in pregnancy, further research is needed to define its true burden, elucidate risk factors, and establish evidence‐based guidelines for diagnosis, empiric therapy, and prevention in affected populations.

## Funding

No funding was received for conducting this study.

## Disclosure

No funds, grants, or other support were received.

## Consent

Written informed consent for publication of the patient’s clinical details and/or clinical images was obtained from the patient. A copy of the consent is available for viewing upon request.

## Conflicts of Interest

The authors declare no conflicts of interest.

## Data Availability

Research data are not shared.
